# Supplementary material for: Effect of academic stress, educational environment on academic performance & quality of life of medical & dental students; gauging the understanding of health care professionals on factors affecting stress: A mixed method study
Source: PLoS One. 2023 Nov 28;18(11):e0290839. doi: 10.1371/journal.pone.0290839 (PMC10683982; doi:10.1371/journal.pone.0290839)

PLOS ONE

“Effect of academic stress, educational environment on academic performance & quality of life of medical & dental students; Gauging the understanding of health care professionals on factors affecting stress: A mixed method study”

--Manuscript Draft--

**Manuscript Number:**	PONE-D-23-12972
**Article Type:**	Research Article
**Full Title:**	“Effect of academic stress, educational environment on academic performance & quality of life of medical & dental students; Gauging the understanding of health care professionals on factors affecting stress: A mixed method study”
**Short Title:**	Academic stress, educational environment, academic performance and quality of life
**Corresponding Author:**	Neelofar Shaheen, MBBS, MHPE, PhD (Scholar)<br>Peshawar Medical College<br>Peshawar, KPK PAKISTAN
**Keywords:**	
**Abstract:**	Introduction: To correlate the effects of academic stress and educational environment on academic performance and quality of life of students and gauge the perspectives of health care professionals on stress.<br>Methods: A mixed method study was conducted from February to May 2022, comprising the students of the Peshawar Medical and Dental College. During the cross sectional survey (Phase , the participants completed the questionnaires comprising of DREEM inventory, Academic Stress Scale, and WHO-QOL Scale. Academic performance was evaluated through last year's annual assessment results of the students. Healthcare professionals’ perspectives on students’ academic stress were also considered.<br>Results: The mean age of the sample (n=440) was 22.24 ±1.4 years. The Cronbach Alpha reliability of the DREEM inventory in our study was 0.877, that of the Academic Stress Scale was 0.939 and WHOQOL was 0.895. On thematic analysis, 112 codes were generated which converged into 9 categories and finally 5 themes emerged which were Challenging educational environment/system, Psychological need and support, Individual differences, Relationship and family life, and Adjustment issues. More than half of the students (n=230, 52.3%) reported better QOL and the majority perceived a positive educational environment (n=323, 73.4%) and higher academic stress (n=225, 51.1%). Males had significantly more academic stress (p<0.05). Those who perceived a positive educational environment and better QOL had better academic performance (p<0.05). Academic performance was positively and significantly correlated with QOL and academic stress (p=0.000).<br>Conclusion: Academic stress leads to poor academic performance and better QOL leads to better academic performance. Students facing multi-dimensional stressors need timely psychological support and the role of the family is very important.<br>Key Words: Academic stress, performance, Educational Environment, Psychological support
**Order of Authors:**	Muhammad Hassan Wahid, MBBS (Scholar)
	Mifrah Rauf Sethi, MphiL, CHPE, MSc., PhD (Scholar)
	Neelofar Shaheen, MBBS, MHPE, PhD (Scholar)
	Kashif Javed, MBBS (Scholar)
	Muhammad Osama, MBBS (Scholar)
	Tariq Firdos, MBBS (Scholar)
	Abdul Ilah, MBBS (Scholar)
	Ijlal Aslam Qazi, MBBS (Scholar)
**Additional Information:**	

Powered by Editorial Manager® and ProduXion Manager® from Aries Systems Corporation

Question	Response
**Financial Disclosure**<br><br>Enter a financial disclosure statement that describes the sources of funding for the work included in this submission. Review the submission guidelines for detailed requirements. View published research articles from *PLOS ONE* for specific examples.<br><br>This statement is required for submission and **will appear in the published article** if the submission is accepted. Please make sure it is accurate.<br><br>**Unfunded studies**<br>Enter: *The author(s) received no specific funding for this work.*<br><br>**Funded studies**<br>Enter a statement with the following details:<br>• Initials of the authors who received each award<br>• Grant numbers awarded to each author<br>• The full name of each funder<br>• URL of each funder website<br>• Did the sponsors or funders play any role in the study design, data collection and analysis, decision to publish, or preparation of the manuscript?<br>• **NO** - Include this sentence at the end of your statement: *The funders had no role in study design, data collection and analysis, decision to publish, or preparation of the manuscript.*<br>• **YES** - Specify the role(s) played.<br><br>* typeset	The author(s) received no specific funding for this work.
**Competing Interests**<br><br>Use the instructions below to enter a competing interest statement for this submission. On behalf of all authors, disclose any competing interests that could be perceived to bias this work—acknowledging all financial support and any other relevant financial or non-financial competing interests.	The authors have declared that no competing interests exist.

This statement is required for submission and **will appear in the published article** if the submission is accepted. Please make sure it is accurate and that any funding sources listed in your Funding Information later in the submission form are also declared in your Financial Disclosure statement.<br><br>View published research articles from *PLOS ONE* for specific examples.<br><br>**NO authors have competing interests**<br><br>Enter: *The authors have declared that no competing interests exist.*<br><br>**Authors with competing interests**<br><br>Enter competing interest details beginning with this statement:<br><br>*I have read the journal's policy and the authors of this manuscript have the following competing interests: [insert competing interests here]*<br><br>* typeset	
**Ethics Statement**<br><br>Enter an ethics statement for this submission. This statement is required if the study involved:<br><br>• Human participants<br>• Human specimens or tissue<br>• Vertebrate animals or cephalopods<br>• Vertebrate embryos or tissues<br>• Field research<br><br>Write "N/A" if the submission does not require an ethics statement.<br><br>General guidance is provided below. Consult the submission guidelines for detailed instructions. **Make sure that all information entered here is included in the Methods section of the manuscript.**	Ethical approval was granted by the Ethical Review Committee of the PRIME foundation and ERC Approval number is Prime/ERC/2022-01

**Format for specific study types**<br><br>**Human Subject Research (involving human participants and/or tissue)**<br>• Give the name of the institutional review board or ethics committee that approved the study<br>• Include the approval number and/or a statement indicating approval of this research<br>• Indicate the form of consent obtained (written/oral) or the reason that consent was not obtained (e.g. the data were analyzed anonymously)<br><br>**Animal Research (involving vertebrate animals, embryos or tissues)**<br>• Provide the name of the Institutional Animal Care and Use Committee (IACUC) or other relevant ethics board that reviewed the study protocol, and indicate whether they approved this research or granted a formal waiver of ethical approval<br>• Include an approval number if one was obtained<br>• If the study involved *non-human primates*, add *additional details* about animal welfare and steps taken to ameliorate suffering<br>• If anesthesia, euthanasia, or any kind of animal sacrifice is part of the study, include briefly which substances and/or methods were applied<br><br>**Field Research**<br><br>Include the following details if this study involves the collection of plant, animal, or other materials from a natural setting:<br>• Field permit number<br>• Name of the institution or relevant body that granted permission	
**Data Availability**<br><br>Authors are required to make all data underlying the findings described fully available, without restriction, and from the time of publication. PLOS allows rare exceptions to address legal and ethical concerns. See the PLOS Data Policy and FAQ for detailed information.	No - some restrictions will apply

Powered by Editorial Manager® and ProduXion Manager® from Aries Systems Corporation

A Data Availability Statement describing where the data can be found is required at submission. Your answers to this question constitute the Data Availability Statement and **will be published in the article**, if accepted.<br><br>**Important:** Stating 'data available on request from the author' is not sufficient. If your data are only available upon request, select 'No' for the first question and explain your exceptional situation in the text box.<br><br>Do the authors confirm that all data underlying the findings described in their manuscript are fully available without restriction?	
**Describe where the data may be found in full sentences. If you are copying our sample text, replace any instances of XXX with the appropriate details.**<br><br>• If the data are **held or will be held in a public repository**, include URLs, accession numbers or DOIs. If this information will only be available after acceptance, indicate this by ticking the box below. For example: *All XXX files are available from the XXX database (accession number(s) XXX, XXX.).*<br>• If the data are all contained **within the manuscript and/or Supporting Information files**, enter the following: *All relevant data are within the manuscript and its Supporting Information files.*<br>• If neither of these applies but you are able to provide **details of access elsewhere**, with or without limitations, please do so. For example:<br><br>*Data cannot be shared publicly because of [XXX]. Data are available from the XXX Institutional Data Access / Ethics Committee (contact via XXX) for researchers who meet the criteria for access to confidential data.*<br><br>*The data underlying the results presented in the study are available*	Data cannot be shared publicly because of confidential academic records of the participants. Data are available from the PRIME Foundation Institutional Ethics Committee (contact via mifrahrauf@gmail.com) for researchers who meet the criteria for access to confidential data.

from (include the name of the third party and contact information or URL).<br>• This text is appropriate if the data are owned by a third party and authors do not have permission to share the data.<br><br>* typeset	
Additional data availability information:	

Powered by Editorial Manager® and ProduXion Manager® from Aries Systems Corporation

Manuscript

Click here to access/download;Manuscript;Acad Stress QOL & Acad Perf (M1).docx

"Effect of academic stress, educational environment on academic performance & quality of life of medical & dental students; Gauging the understanding of health care professionals on factors affecting stress: A mixed method study"

Abstract

Introduction: To correlate the effects of academic stress and the educational environment on academic performance and quality of life of students and to gauge the perspectives of health care professionals on stress.

Methods: A mixed-method study was conducted from February to May 2022, comprising students from the Peshawar Medical and Dental College. During the cross sectional survey ([illegible]ase , the participants completed the questionnaires comprising of DREEM inventory, Academic Stress Scale, and WHO-QOL Scale. Academic performance was evaluated through last year's annual assessment results of the students. Healthcare professionals' perspectives on students' academic stress were also considered.

Results: The mean age of the sample (n=440) was 22.24 ±1.4 years. The Cronbach Alpha reliability of the DREEM inventory in our study was 0.877, that of the Academic Stress Scale was 0.939 and WHOQOL was 0.895. In thematic analysis, 112 codes were generated which converged into 9 categories, and finally 5 themes emerged that were challenging educational environment / system, psychological need and support, individual differences, relationship and family life, and adjustment issues.

More than half of the students (n=230, 52.3%) reported better QOL and the majority perceived a positive educational environment (n=323, 73.4%) and higher academic stress (n=225, 51.1%). Males had significantly more academic stress ($p<0.05$). Those who perceived a positive educational environment and better QOL had better academic performance ($p<0.05$). Academic performance was positively and significantly correlated with QOL and academic stress ($p=0.000$).

Conclusion: Academic stress leads to poor academic performance and better QOL leads to better academic performance. Students facing multidimensional stressors need timely psychological support,, and the role of the family is very important.

Keywords: Academic stress, academic performance, educational environment, psychological support

Introduction

Academic stress is defined as a body's response to academic-related demands that exceed the adaptive capability of students.[1] According to a survey carried out by the team of 'Dictionary of Human Geography' in the U.S.A, academic stress is more often seen in medical and dental students as the total prevalence of stress in medical and dental students was reported as 63% and the prevalence of severe stress was 25% which affected their quality of life and performance directly.[2] High academic stress among medical and dental students is a phenomenon that transcends socio-cultural factors, economic status and course patterns.[3] The higher demands associated with academic studies may interfere with demands in other domains of life and vice versa.[4] Mental health is especially affected by perceived stress among medical and dental students from aged-matched non-medical student peers. Medical and dental students experience high cynicism and high emotional exhaustion due to work overload.[5]

Academic self-efficacy, perceived stress and preferred learning styles have been linked to academic performance and have a significant effect on medical and dental college students.[6] Perceived academic stress affects one's quality of life. Quality of life is an overarching term for the quality of various domains in life, therefore, it is a subjective multidimensional concept that defines a standard level for emotional, physical, mental, and social well-being. It is believed that for one's proper efficiency in work and good mental and physical status, one must possess a good quality of life.[7] Students who disclose utilizing a multitude of self-care practices throughout their undergraduate journey in medical and dental colleges reported a decrease in the strength of the relationship between perceived stress and quality of life.[8]

Medical schools can produce intense psychological distress in their students. However, physical exercise, sufficient leisure time and a good lifestyle can help students to be potential leaders in the field of health.[9] Quality of life is an essential component of learning and has strong links to the

practice and study of medicine and dentistry. There is burgeoning research to suggest that medical students are experiencing mental and psychological troubles such as perceived academic stress and burnout.[10] Acute academic stresses are positive and enable a person to perform better from the start of the career, but the chronic form is the silent killer. No one is shielded from stress, but it is more prevalent in medical and dental students. The major contributing factors are an unbalanced lifestyle and study overload.[11]

Mental and psychological QOL scores were lower among medical students than among the age-matched general population. The prevalence of stress symptoms was similar regardless of race, colour, and cast. The students had to struggle hard to maintain their academic performance and had been having little physical activity and disturbed sleep patterns.[12] Perception about the educational environment of the institution was more positive than negative among medical students. Better performance in examinations was associated with better academic self-perception and social self-perception in students.[13]

Based on this evidence, the present study aims to determine the effect of academic stress, and educational environment on QOL and academic performance of medical and dental students. As every place have its challenges and circumstances, this study also planned to gauge the understanding of healthcare professionals' perspective (qualitatively) on factors relevant to the academic stress of students.

Methodology

This sequential-explanatory mixed-method study was conducted on medical and dental students of a private institution in Peshawar, Pakistan from February 2022 to May 2022. The participants were recruited in March 2022. The age range was between 19 to 25 years. All the students above the age of 18 years were included in the study while students with mental health issues and 1st-year medical students were excluded from the study. The authors had access to the demographic data of the participants and individual participants could be identified during or after data collection.

During Phase 1 (Quantitative part) structured, reliable and validated questionnaires were distributed among the medical and dental students. In phase 2 (Qualitative part) interviews were conducted with healthcare professionals to explore their perspectives on the factors they considered relevant to the student's academic stress.

PHASE 1: A cross-sectional survey was conducted including all medical and dental students from 2nd to final year, including the purposive sampling technique. Ethical approval was taken from the Ethical Review Committee of Prime Foundation (PRIME/ERC/2022-01), Pakistan, and permission was obtained from the concerned authorities (Vice-chancellors/Dean) of the institutions before the start of data collection. All the students who participated in the study were informed about the objectives of the study and written informed consent was obtained. Demographic data were collected on structured questionnaires before collecting the data, followed by applying the following scales.

INSTRUMENTS:

World Health Organization, Quality of Life Scale: (WHO-QOL):

The World Health Organization Quality of Life (WHOQOL-BREF) was used to assess the quality of life, which is an empirical instrument to assess QOL. It comprises 26 items and measures the

domains of physical health; psychological health; social relationships; and environment in addition to measuring general QOL. Responses to questions are on 5 points Likert scale where “1” represents "disagree" or "not at all" and “5” represents "completely or extremely agree". Higher scores indicate better quality of life and vice versa.[16]

Academic Performances:

Academic performance is the output of one’s learned particular curriculum which involves assessment through tests from a particular syllabus. Annual professional exam results were taken as a measure of academic performance. The grading scale used was 1. (10-35%), 2. (35-60%), 3. (60-85%), and 4. (85-100%).

Dundee Ready Educational Environment Measure Inventory:

The 50-item DREEM questionnaire is scored on a five-point Likert scale, ranging from 0 = strong disagreement to 4 = strong agreement. The overall total ranges from 0 to 200. Of the 50 items, 9 are negatively stated items and reverse scored items. The questionnaires have been divided into five subscales: students' perception of learning (SPL), students' perception of teachers (SPT), students' academic self-perceptions (SAP), and student's perception of the atmosphere (SPA), and students' social self-perception. For the DREEM subscales, the maximum scores are 48, 44, 32, 48 and 28, respectively, the Practical Guide described by McAleer and Roff for interpreting the overall and subscale scores. [17, 18]

Academic Stress Scale:

The 40-items academic stress scale was originally developed by Kim (1970). Five-point Likert scale ranging from “no stress” to “extreme stress”. The scale was classified into five sub-areas containing 8 items each: personal inadequacy, fear of failure, interpersonal difficulties with teachers, teacher-pupil relationship/teaching methods and inadequate study facilities. The total number of items was 40. Therefore 160 (4 into 8) is the maximum possible score and the highest score for each factor would be 32.[19]

PHASE 2:

This was followed by in-depth qualitative interviews with 10 healthcare professionals from different departments of medical sciences. Healthcare professionals were eligible for qualitative interviews with the age range of 30 to 65 years. Professionals were purposively selected to reflect the diversity of perspectives and educational attainment. The primary investigator interviewed the health care professionals to explore their perspective on the factors they considered relevant to the student's academic stress, lasting for 40-60 minutes, transcribed into English verbatim and independently validated for coding reliability. The interview guide was developed by following the protocol for questionnaire development and was validated by the experts.

Data Analysis (Quantitative phase)

The analysis of the data was carried out using SPSS version 25. Basic variables were analysed using descriptive statistics for finding frequencies and percentages, means, and standard deviation. The internal consistency of the scales was measured through Cronbach's Alpha reliability and a value equal to or greater than 0.70 was considered satisfactory. The Chi-square test was used to find the differences between demographic variables, academic stress, performance, QOL and educational environment. In Addition, the Pearson correlation test was used to check the relation between all four components (stress, QOL, performance and environment). The results of all the tests of significance were considered significant at $p < 0.05$ level.

Data Analysis (Qualitative Phase)

For the qualitative part, we used thematic analysis using Virginia Braun and Victoria Clarke six steps approach to generate themes from the qualitative data. The six steps included familiarization with the data, generating initial codes, searching for themes followed by reviewing, Understanding, naming the themes, and finally producing the results.[20] To ensure the quality of qualitative data we applied triangulation and member-checking methods.

Results

A total of 500 students from the private institutes of medical and dental college were asked to participate and the response rate was 80% (440). The mean age of the population was 22.24±1.5 years with the age range from 18 to 25 years. The majority were male students (n=224, 50%), who lived in the nuclear family system (n=274, 62.3%). The Cronbach alpha reliability of the DREEM inventory was 0.877, and that of the Academic Stress Scale and WHOQOL was 0.939 and 0.895 respectively. More than half of the students (n=230, 52.3%) reported better QOL, the majority perceived a positive educational environment (n=323, 73.4%) and around half of them had academic stress (n=225, 51.1%). Considering last year's annual examinations percentages of students as a tool for measuring academic performance, more than half of the students (n=387, 88%) got 60% to 85% marks and were labelled as better performers as mentioned in Table 1.

Males had significantly more academic stress ($p<0.05$) as compared to females. Those students who perceived a positive educational environment and better QOL had better academic performance ($p<0.05$), as shown in Table 2.

The Pearson correlation showed a significant moderate but negative correlation between academic performance and academic stress and similarly showed a significant moderate but positive correlation with the quality of life. In addition, the results showed a non-significant negative correlation with the educational environment. Whereas the quality of life has a significantly moderate but negative correlation between academic stress and educational environment, but a significantly moderate and positive correlation between the educational environment and academic stress, as shown in Table 3.

Table 1: Basic details of demographic variables (n=440)

S. No	Variables		n (%)
1	Gender	Male	224(51%)
		Female	216(49%)
2	Speciality	MBBS	336(76.4%)
		BDS	104(23.6%)
3	Family Structure	Joint family	166(37.8%)
		Nuclear family	274(62.2%)
4	Year of Study	Pre-clinical	213 (48.4%)
		Clinical	227 (51.6%)

5	Academic Performance	10% to 35%	0
		35% to 60%	40(9%)
		60% to 85%	387(88%)
		85% to 100%	13(3%)
6	WHOQOL Scale	Better QOL	230 (52.3%)
		Poor QOL	210 (47.7%)
7	DREEM Inventory	Excellent environment	26 (5.9%)
		More positive than negative environment	323 (73.4%)
		Plenty of problems	91 (20.7%)
8	Academic Stress scale	Higher academic stress	225 (51.1%)
		No academic stress	215 (48.9%)

Table 2: Chi-square test between demographic variables (gender & academic performance) with DREEM Inventory, WHOQOL and academic stress scale (n=440).

**Variables**		**Male n (%)**	**Female n (%)**	**X^2 (P- value)**	**35%-60%**	**60%-85%**	**85%-100%**	**X^2 (p-value)**
DREEM Inventory	Excellent	11 (4.9%)	15 (6.9%)	2.33 (.312)	5 (20.8%)	19 (5.1%)	2 (4.8%)	20.3 (.002)
	Positive	161 (71.9%)	162 (75%)		16 (66.7%)	267 (72.2%)	38 (90.5%)	
	Plenty of Problems	52 (23.2%)	39 (18.1%)		2 (4.8%)	38 (90.5%)	2 (4.8%)	
WHOQOL	Better QOL	112 (50%)	118 (54.6%)	.945 (.331)	8 (33.3%)	190 (51.4%)	29 (69%)	**9.14 (.027*)**
	Poor QOL	112 (50%)	98 (45.4%)		16 (66.7%)	180 (48.6%)	13 (31%)	
Academic Stress	High Stress	125 (55.8%)	100 (46.3%)	**3.98 (.046*)**	11 (45.8%)	195 (52.7%)	15 (35.7%)	**8.45 (.038*)**
	No Stress	99 (44.2%)	116 (53.7%)		13 (54.2%)	175 (47.3%)	27 (64.3%)	

Table 3: Pearson Coefficient Correlation between academic performance, academic stress scale, WHOQOL scale and DREEM Inventory (n=440).

Measure	Academic Performance	Academic Stress Scale	WHOQOL Scale	DREEM Inventory
**Academic Performance**	1			
**Academic Stress Scale**	-0.096* (0.044)	1		
**WHOQOL Scale**	0.103* (0.031)	-0.097* (0.043)	1	
**DREEM Inventory**	-0.037 (0.438)	0.338** (0.000)	-0.285** (0.000)	1

Qualitative Results:

We used an inductive thematic analysis approach to code the transcripts. The transcripts were read in an iterative pattern by the researchers and manually coded. Around 112 codes emerged in the first cycle of coding which converged into 9 categories of codes. The categories with mutually exhaustive meanings were converted into 5 themes.

Theme 1: Challenging Educational Environment:

Most Healthcare professionals (n=8, 80%), reported that medical and dental students are often exposed to challenging educational environments. Medical students have to fulfil multiple tasks in a short span of time and sometimes in the emotionally stressful situations of dealing with patients in pain. Such circumstances lead to perceived academic stress and affect the academic performance of the students.

> *Students have to fulfil multiple tasks along with dealing with the patients in a short time period, in which they have to achieve their targets in educational settings. (G=8).*

However, two interviewees were of the opinion that a challenging educational environment makes the students strong and they perform well.

> *Challenging Educational Environment is useful in the sense that it makes people strong. (G=2)*
>
> *Challenging Educational Environment leads to better performance under stress. (G=7)*

Theme 2: Psychological needs and support

All the interviewees (n=10, 100%) thought that early psychological assessment and counselling are very important for students and should be made a core component in the early years of a student's life at-college.

For every student, it's essential to have an early psychological assessment and access to a counsellor to discuss their problems and stressors and to help them cope with their problems. (G=10)

Theme 3: Individual differences

The majority of the interviewees (n=9, 90%) were of opinion that students differ from each other concerning personality traits and individual characteristics, so they should be facilitated accordingly. The role of mentorship is extremely important for the better mental health performance of the students.

> *Students are different from each other, in every aspect, even having different personality traits that reflect in their appearance and behaviour. They face different sorts of circumstances during their course of studies. A single factor cannot affect all students in the same manner. (G8)*

However, one participant was of the opinion that in this age group, almost all the students are affected similarly as their challenges are somewhat the same.

> *Almost all the students are of the same age and face the same challenges, which lead them to academic stress and poor academic performance. (G8)*

Theme 4: Social and Family relationships

Most of the interviewees (n=8, 80%) believed that social life and relationships are very important for students. A supportive parent and sibling relationship help the students to develop a positive way of thinking leading to a balanced life, less academic stress, and better performance.

> *The role of social and family relationships is very important in terms of a better quality of life and academic performance. A strong supportive home environment and family can reduce academic stress. (G8)*

However, two of the healthcare professionals narrated that social relationships have little importance in the life of medical students as they don't have time to maintain them.

Medical and Dental students are very busy in their everyday routines, so their social relationships affect them little. (G3)

Medical and Dental students can't maintain their social life at the expense of their precious time, so social relationships don't affect them significantly. (G9)

Theme 5: Adjustment issues

Most of the interviewees (n=9, 90%) reported that newly admitted students have to face adjustment issues in medical schools and especially in the hostels because of change in the environment. These early stressors affect the students' quality of life and ultimately their academic performance. Students should be given some window period to adjust to a new environment and the proper guidance from seniors and teachers is very important in helping them to adjust to the new environment.

Many students face adjustment difficulties at the start of the academic period, because of homesickness, along with roommates, so it's quite difficult for them and it affects their QOL and also their performance. (G9)

It should be mandatory and important to give the space, a lively and friendly environment to the students to adjust to this challenging environment. (G10)

However, one of the interviewees believed students have already experienced hostel life and long study hours during their entry test preparations and intermediate levels, so the adjustment to a new environment is not a big deal.

Students have already gone through such situations before like most of them have experienced hostel life and studied for more than 15 hours a day that's why they are in medical college. (G6)

Discussion

This study is an attempt to draw attention to the effects of the educational environment, and academic stress on the academic performance and quality of life of students and also gauge the perspectives of healthcare professionals on the stress levels of students in a tough educational environment. The reliability of scales, used in this study, is internally consistent and reliable. The reliability of the World Health Organization quality of life scale (WHOQOL) is in line with other studies, ranging from 0.80 to 0.86.[21-23] The reliability of the DREEM inventory (0.877) in our study was slightly less than the reliability of the same tool found in other studies (0.925).[24]

In our study, the majority of the participants perceived the educational environment as positive (73.4%), which is in alignment with other studies.[25-28] The results of our study regarding the high prevalence of academic stress in medical and dental students have been supported by the study conducted in the USA and at King Saud University, KSA where the prevalence of academic stress among students was 30% and 63%, respectively, affecting the academic performance of the students.[1,2] In our study, males had more academic stress than females which is contrary to the results of another study, in which there was no significant gender difference.[11] A systematic review identified 23 studies that evaluated the relationship between academic assessment and psychological distress among female medical students which is in line with our research findings.[29]

Our study results found no significant differences in opinion among male and female students in terms of DREEM inventory which is in line with few studies that report insignificant gender differences in opinion regarding the educational environment.[33-36] However, these findings are in contrast with few studies, in which female students perceived the educational environment as more positive than male students.[30-32]

The correlation between academic stress and quality of life has been aligned with the findings of Garg K et al, in North India.[3] Whereas a better quality of life leads towards better academic performance and vice versa and the importance of a properly balanced life for better academic performance and better mental health has been supported by many recent studies carried out in

different parts of the world.[7,9.10,12] Academic stress was significantly correlated with the educational environment which is in line with the findings of another study.[37] Similarly, a systematic review and a survey carried out at Islamia University Bahawalpur, reported a negative association between academic stress and QOL among university students, which is in accordance with our research findings.[38,39]

In our study, students of pre-clinical and clinical years have perceived an equal amount of academic stress which is in contrast to the findings of another study, which found a high prevalence of stress in students of pre-clinical years.[11] Similarly, the findings of a study conducted in Ghana confirmed that the students in senior high schools with a pleasant physical environment perform better which is contrary to our research findings.[40]

The association between academic stress and QOL with academic performance in our study has been supported by other studies.[10,14] Whereas, the correlation between academic performance and educational environment in our study is contradictory to the findings of another study conducted in Pakistan, in which a better perceived educational environment leads to better academic performance.[13]

In our study, the majority of the students (74%) perceived the educational environment better and had better QOL (62%) which is contrary to many other studies across the globe in which medical and dental students report average quality of life and perceive the educational environment as negative.[2,3,7,14]

The most important verbalism of our qualitative part; lack of time, the imbalance between social and academic life, distressful daily situations in college and hospital environments and individual personality differences of students has been supported by many different studies.[4-6, 10] Extensive courses and training in medical and dental studies lead towards a change of psychological and physical QOL, which has been supported by a few studies.[8, 12]

Our studies showed no significant effect of family structure on students' academic performance which is aligned with the Azumah FD study's results and contradicts Hanul P's study, which shows

a significant effect of family structure.[41, 42] Family support and good relationships are very important for better academic performance as per our qualitative result which is similar to a study carried out in Bangladesh.[43]

A study conducted in nursing college reported that work overload and academic stress lead to a challenging educational environment leading to burnout of students' psychological and mental health, supporting our qualitative theme of a challenging educational environment in the medical career leading to psychological distress.[15]

Conclusion

The study concluded that most of the students are under high academic stress, because of the challenging educational environment, lack of time, and imbalance between social and personal life. Academic stress leads to poor academic performance and affects the QOL of the students,

however, the educational environment has a non-significant relationship with academic performance. The role of parents, family and mentorship is very important for better mental health and academic performance. Early psychological assessment, support and Counseling should be made a part of the routine activities of students at college.

Limitations

The main limitation of our study is that we have taken two institutes only because of the time limit and feasibility of the research. Apart from that our sample size is a bit small, so in the future will focus on the longitudinal type of research with a large sample size.

Acknowledgements

We are highly grateful to all the students who took a keen interest in giving data. We are grateful to the healthcare professionals and the administration of the private institutes for encouraging, supporting and helping us in the completion of our research.

Declaration of Interest

The authors declare no conflict of interest.

References

1. Derek G, Rott J, Michale W. Quality of life. [Internet](Dictionary of Human Geography), (ed.5); Wiley Blackwell. (cited 28 April 2022). Available from: https://www.academia.edu/39335132/Human_Geography_5th_Edition_Edited_by. ISBN 978-14
2. Hamza MA, Ebrahim SM, Gominda P. Alfaris E. Stress and its effects on medical students:a cross-sectional study. J Health popul Nutr. 2017; 29(5): 516-22. doi:10.3329/ghpn.v2915.8906.
3. Garak AM, Kumar DP. Stress among medical students: A cross-sectional study from a Norin Indian Medical University. Indian J Psychiatry. 2017; 5914:502-04. doi:10.4103/psychiatry. Indian J Psychiatry 239 /17.

4. Bergmann C, Muth T, Loerbroks A.Medical students perception of stress due to academic studies and its interrelationships with other domains of life: a qualitative study. Med Educ Online. 2019; 24(1): 1-6. doi:10.1080/10872981.2019.1603526.
5. Mckerrow L, Patricia A, Caretta H, Juve A. Trends in medical students' stress, physical and emotional health throughout training. Med Edu Online. 2020: 25(1); 1-7. doi.10.1080/10872981.2019.1709278.
6. Oleg NM, Christian UK, Richard JS. Rasch analysis of the Perceived Stress Scale: Transformation from an ordinary to a linear measure. J Health Psychol. 2019; 24(8):1070-81. doi.10.11.77/EPUB 2017 Jan 30.
7. Alsulami S, Omar Z, Haseeb M, Mohammad S Binnwejim. Perception of academic stress among health science, Preparatory Program in two Saudi Universities. Adv Med Educ Pract. 2018: 9; 159-64. doi:10.2147/ Amep. S143151.eCollection 2018.
8. Erin EA, Jeffery SW, Ryan DJ, Hyacinth RCM. Medical students who engage in self-care report less stress and higher quality of life. BMC Med Educ. 2018; 18(1): 189. doi:10.1186/s12909-018-1296-X.
9. Dayna MY, Christopher AF, Mark DS. Effects of group fitness classes on stress and quality of life of medical students. J Am Osteopath Assoc. 2017; 117(11): 17-25. doi: 10.7556/jaoa.2017.140.
10. Marcus AH, Christian UK, Susan JH. The quality of life of medical students studying in Newzeland: a comparison with non-medical students and a general population reference group. Teach Learn Med. 2016; 24(4): 334-40. doi:10.1080/10401334
11. Latha G, Smriti S. Prevalence and causes of stress in newly joined medical and dental students varies with gender. Int J physiol 2019; 7(1): 46-50. doi10.37506/igop.v7/1.263.
12. Liselotten D, Mathew RT, Tait DS. Systemic review of depression, anxiety and other indicators of psychological distress U. S. and Canadian medical students. Acad Med Journal. 2018; 81(4): 20-27. doi:10.109770000 1888-4449.
13. Sarwar S, Tarique S. Perception of educational environment: Does it impact academic performance of medical students? J Pak Med Assoc. 2016; 66(10): 1210-14.PMID:27686291
14. Rehman R, Ghias K, Fatima S, Hussain M, Faiza A. Dream of a conductive learning environment: One DREEM for all medical students. J Pak Med Assoc. 2017; 67(1): 7-11.PMID:28065945
15. Peyton SR, Anderson KW, Mayer K.Nursing Faculty Professional Quality of Life and Resilience in a Changing Educational Environment. J Nurs Educ. 2022; 61(6): 330-33.doi:10.3928/01484834-20220404-12

16. Skevington S, Lotfy M, O'Connell K. The World Health Organization's WHOQOL-BREF quality of life assessment: Psychometric properties and results of the international field trial. A report from the WHOQOL Group. Qual Life Res. 2004.13: 299–310.
17. Prashanth GP, Ismail SK. The Dundee Ready Education Environment Measure: A prospective comparative study of undergraduate medical students' and interns' perceptions in Oman. Sultan Qaboos Univ Med J. 2018; 18(2): e173-81. doi: 10.18295/squmj.2018.18.02.009.
18. McAleer S, Roff S. A practical guide to using the Dundee Ready Education Environment Measure (DREEM). In: Genn JM, editor. Curriculum, Environment, Climate, Quality and Change in Medical Education: a Unifying Perspective. AMEE Education Guide No 23. Dundee, UK: Association for Medical Education in Europe; 2001:29–33.
19. Student Academic Stress Scale website. [Online], 2020. Available from: http://shodhganga.inflibnet.ac.in/bitstream/10603/8093/14/14_appendices.pdf
20. Braun V, Clarke V, Hayfield N & Terry G. Thematic Analysis. In Pranee Liamputtong (ed.): Handbook of Research Methods in Health Social Sciences. Springer Singapore. 2019. 843-60.
21. Hettiarachchi M, Fonseka CL, Gunasekara P, Jayasinghe P, Maduranga D. How does the quality of life and the underlying biochemical indicators correlate with the performance in academic examinations in a group of medical students of Sri Lanka? Med Educ Online. 2014; 19: 22772.
22. Skevington SM, Lotfy M, O’Connell KA. The World Health Organization’s WHOQOL-BREF quality of life assessment: psychometric properties and results of the international field trial. A report from the WHOQOL group. Qual Life Res. 2004; 13(2):299–310.
23. Krageloh C, Henning M, Hawken S, Zhao Y, Shepherd D, Billington R. Validation of the WHOQOLBREF quality of life questionnaire for use with medical students. Educ Health. 2011;24(2): 5
24. Vaughan B, Carter A, Macfarlane C. *et al.* The DREEM, part 1: measurement of the educational environment in an osteopathy teaching program. BMC Med Educ. 2014; 14, 99-110. doi.org/10.1186/1472-6920-14-99
25. Imran N, Khalid F, Haider II, Jawaid M, Irfan M, Mahmood A, Ijlal Haider M, Sami-ud-din. Student's perceptions of educational environment across multiple undergraduate medical institutions in Pakistan using DREEM inventory. J Pak Med Assoc. 2015; 65(1): 24-8. PMID: 25831669.
26. Aghamolaei T, Fazel I. Medical students' perceptions of the educational environment at an Iranian Medical Sciences University. BMC Med Educ. 2010; 10: 87.
27. Lokuhetty MDS, Warnakulasuriya SP, Perera RIR, De Silva HTR, Wijesinghe HD. Students' perception of the educational environment in a Medical Faculty with an innovative Curriculum in Sri Lanka. South-East Asian J Med Educ. 2010; 4: 9-16

28. Nahar N, Talukder MHK, Khan MT, Mohammad S, Nargis T. Students' Perception of Educational Environment of Medical Colleges in Bangladesh. Bangabandhu Sheikh Mujib Med Uni J. 2011; 3: 97-102.

29. Lyndon MP, Strom JM, Alyami HM, Yu TC, Wilson NC, Singh PP, Lemanu DP, Yielder J, Hill AG. The relationship between academic assessment and psychological distress among medical students: a systematic review. Perspect Med Educ. 2014; 3(6): 405-18. doi: 10.1007/s40037-014-0148-6.

30. Till H. Climate studies: can students' perceptions of the ideal educational environment be of use for institutional planning and resource utilization? Med Teach. 2005; 27(4):332–7.

31. Al-Naggar RA, Abdulghani M, Osman MT, et al. The Malaysia DREEM: perceptions of medical students about the learning environment in a medical school in Malaysia. Adv Med Educ Pract. 2014;5: 177–84. doi:https://doi.org/10.2147/AMEP.S61805

32. Jacobson U, Danielsen N, Edgren G. Psychometric evaluation of the Dundee ready educational environment measure: Swedish version. Med Teach. 2011; 33(5):e267–74.

33. Hammond SM, O'Rourke M, Kelly M, Bennett D, O'Flynn S. A psychometric appraisal of the DREEM. BMC Med Educ. 2012; 12:2.

34. Bassaw B, Roff S, McAleer S, et al. Students' perspectives on the educational environment, Faculty of Medical Sciences. Trinidad Med Teach. 2003; 25(5): 522–6.

35. Al-Hazimi A, Al-Hyiani A, Roff S. Perception of the educational environment of the medical school in King Abdul Aziz University. Saudi Arabia Med Teach. 2004; 26(6):570–3.

36. Payne LK. Comparison of students' perceptions of educational environment in traditional vs. accelerated second degree BSN programs. Nurse Educ Today. 2013; 33(11):1388–92.

37. Chew QH, Holmboe E, Sim K. Learning environment, stress and coping in psychiatry residents within a national training program: a longitudinal study. Perspect Med Educ. 2019; 8(3): 160-6. doi 10.1007/s40037-019-0512-7.

38. Caro JS, Eduardo N. Stress and Quality of life Among University Students: A systematic review. Health Professions Education. 2018; 4: 70-7 doi. 10.1016/j.hpe.2017.03.002

39. Iqbal S, Akram M, Mushtaq I. Relationship between stress and Educational Performance of University Students. REAL. 2021; 4(4): 805-11. doi. https://doi.org/10.47067/real.v4i4.198

40. Baafi RKA. School Physical Environment and students' Academic Performance. Adv Phys Educ. 2020; 10: 121-37. doi:10.4236/ape.2020.102012

41. Azumah FD, Samuel K, Onzaberigu NJ. Effect of Family Structure on Academic Performance of Children. Int J Soc Sci. 2018; 6(10):11-22

42. Hanul P, Lee KS. The association of family structure with health behavior, mental health and perceived academic achievement among adolescents. BMC Public Health. 2020; 20,510. doi. 10.1186/s12889-020-08655-2

43. Islam N, Chakrabarty S. A study of the Relationship between Familial Factors and Academic Motivation and Achievement in High School Students. Psychol Res. 2020. 65(1):87-96. doi:10.1007/s12646-019-00500-7

Figure

Click here to access/download;Figure;Acad Stress perf QOL I1.tif

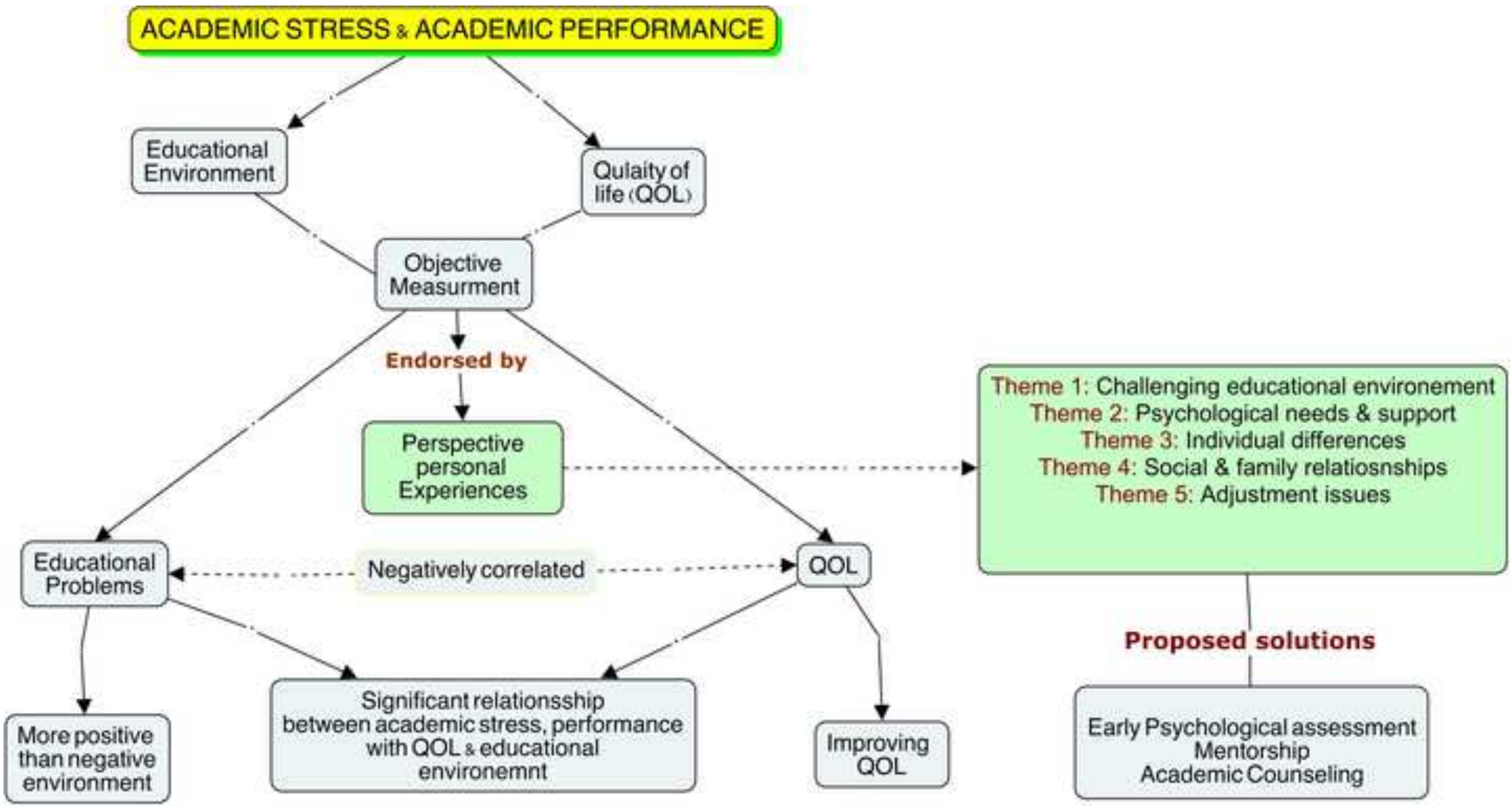

Supplement: Attachment — Submitted filename: PONE-D-23-12972_reviewer.pdf [file pone.0290839.s003.pdf]
