# Supplementary material for: Effect of academic stress, educational environment on academic performance & quality of life of medical & dental students; gauging the understanding of health care professionals on factors affecting stress: A mixed method study
Source: PLoS One. 2023 Nov 28;18(11):e0290839. doi: 10.1371/journal.pone.0290839 (PMC10683982; doi:10.1371/journal.pone.0290839)
Supplement: Attachment — Submitted filename: Response to Reviewers D-23-12972.docx [file pone.0290839.s004.docx]

**Rebuttal Letter: Article No: [PONE-D-23-12972]**

**“Effect of academic stress, educational environment on academic performance & quality of life of medical & dental students; Gauging the understanding of health care professionals on factors affecting stress: A mixed method study”**

Dear Editor,

We would like to express our gratitude to the esteemed reviewers for their reviews and generous consideration in advancing the study further. We have diligently endeavoured to incorporate all the recommendations put forth by the reviewers in our manuscript. Below, you will find a comprehensive response to each of the comments of the reviewers.

**Reviewer #1:** 
**Abstract**

**Comment:** P2L39: The authors wrote" (Phase" , but it is a pair")" is missing.

**Response:** The study had 2 phases so this has been corrected to clarify and also highlighted in the manuscript (L53)

**Comment:** P2L54: The space is still painted yellow.（Before brackets）
**Response:** This mistake has been corrected and highlighted in the manuscript.

**Comment:** P9 Table2: p-value between DREEM and academic performance is .002 that is <0.05, but there is no *.
**Response:** The * has been placed and highlighted. (Table 2)

**Comment:** The subjects of this study were a mix of medical and dental school students. Were there any special characteristics of each school?

**Response:** These were two sister institutes for MBBS & BDS under one organization. The overall environment in the institutes is similar because similarities in administration and students face similar kind of challenges whether they belong to MBBS or BDS. So it was better to have both institutes together in one study.

**Comment:** The authors utilized a mixed methods study in this paper, but the purpose of the qualitative study in particular is unclear.

**Response:** The purpose of the qualitative study is to explore the individual in-depth perspective of experienced faculty (the health care professionals) in detail about all the circumstances they experience during their daily dealings with students regarding academic stress and educational environment. The faculty also highlighted certain factors highlighted by the students in the quantitative phase to elaborate in more detail as it is intended in the sequential exploratory design.

Figure 1 short description has been added to elaborate the key points (L369-371).

**Comment:** Are healthcare professionals appropriate to consider their perspectives on student's academic stress?

**Response:** The healthcare professionals were the faculty members in various departments who had experience dealing with the students to help them in coping with stressful situations during their academic journey. The point has been added and highlighted in the manuscript (L57-58).

**Comment:** It seems to me that the results from the qualitative study are rarely reflected in the discussion and conclusions. We would like to see a more complete discussion of the results obtained in this study, as it is limited to mentioning the agreement or disagreement with previous studies.

**Response:**

The discussion section has been addressed to address the comment. The changes have been highlighted in the manuscript.

**Reviewer #2:**

**Comment:** Line 35 - 37: Abstract did not highlight the research gap the study is trying to address.

**Response:** The introduction of the abstract has been updated to include the research gap. The changes have been highlighted in the manuscript. (L47-51))

**Comment:** Line 39: Phase?

**Response:** The study had 2 phases, so this has been corrected to clarify and highlighted in the manuscript (L53-56).

**Comment:** Line 62 103 (Introduction): Introduction describes previous study findings but did not highlight existing problem fuelling the motivation to conduct the present study.

**Response:** The introduction has been updated and changes highlighted in the manuscript.

(L130-139).

**Comment:** Line 71-72: Please include citation.

**Response:** The citation has been highlighted (L102)

**Comment:** Line 76: Please include a citation.

**Response:** The citation has been highlighted (L107)

**Comment:** Line 93-94: Please include citation.

**Response:** The citation has been highlighted (L126-127)

**Comment:** Line 119: Please justify the decision to exclude first year medical students.

**Response:** First-year medical and dental excluded mainly because of two reasons

1. As per the study protocol, the measure of academic performance was taken as scores of previous professional examinations, which was not possible in the case of newly admitted students

2. As participants were recruited in February 2022 and the first-year students had joined the institutes in January so students did not face academic stress yet.

The justification has also been added briefly in the manuscript and highlighted.

**Comment:** Line 135: In the result section, the authors reported WHOQOL score categorically as Better vs. Poor QOL. Please indicate the cut-off point use to determine whether the participants belong to either better or poor QOL.

**Response:** The following line has been added in the instrument section and highlighted in the manuscript

“Higher scores based on the mean average indicate better quality of life and vice versa”.

**Comment:** Line 146: Suggestion: It is recommended for authors to indicate what does this range of score suggests e.g., Very low, Low, High, Very high.

**Response:** The amendments have been done as per the suggestion and changes are highlighted in the manuscript.

**Comment:** Line 154-156: Please recheck sentence structure. Please elaborate how the scores were computed or the cut-off score to determine if participants belong to the excellent/ more positive than negative environment/ plenty of problems category.

**Response:** The sentence has been restructured for clarity and highlighted in the manuscript

(L184 -187)

**Comment:** Line 157: Please indicate the cut-off score for no vs. high academic stress.

**Response:** The following line has been rephrased and highlighted in the manuscript

The 40-items academic stress scale was originally developed by Kim (1970). Five-point Likert scale ranging from “no stress i.e., 0” to “extreme stress i.e., 4”.

**Comment:** Line 162: Not sure what the phrase "(4 into 8)" means in this sentence.

**Response:** There were 8 items in each of the 5 subscales. Each item is scored from “no stress i.e., 0” to “extreme stress i.e., 4”. So, the maximum possible score was 4x8=32. The sentence has been rephrased to make it easy to comprehend and highlighted in the manuscript.

**Comment:** Line 166: Please justify sample size estimation for the qualitative interviews. Who are these healthcare professionals... are they nurses, physicians, dietician?

**Response:** For qualitative research, purposive sampling lets us select the participants who can give a deep insight into the subject matter of concern so even a small sample size is enough as long as their detailed perspectives fulfil the objectives of the research. The sample size is also governed by saturation, i.e., there comes a point when the same information or perspective is repeated by the interviewees at that point, the researchers can stop taking interviews at the point of saturation. Both these rules were followed by the researchers during qualitative data collection.

The healthcare professionals were basically the faculty members from various departments of the institutes under study who had experience as counsellors of the students and could give a deeper insight into various challenges faced by the students during their stay at medical or dental college.

**Comment:** Line 180-182: Please indicate the r coefficient value for weak, moderate or strong correlation.

**Response:** The following line has been added and highlighted in the manuscript. The (r_s_) is mentioned because the Pearson correlation is replaced by the Spearman correlation as indicated by the comments of the reviewers

“To check the strength of association, the values of (r_s_) are 0-0.19 as weak, 0.40-0.59 as moderate, 0.6-0.79 as strong and 0.8-1 as very strong correlation”.

**Comment:** Line 191: Please amend to (N = 440)

**Response:** The issue has been addressed and highlighted in the manuscript.

**Comment:** Line 200 - 202: Please address the academic performance of students who have different level of stress as indicated by Table 2.

**Response:** The issue has been addressed and highlighted in the manuscript.

**Comment:** Line 203 - 209: Instead of highlighting the direction of correlation, i.e., positive/negative, it is suggested for author to directly explain what does the negative/positive correlation means. For example, the sentence "The Pearson correlation showed a significant moderate but negative correlation between academic performance and academic stress and similarly showed a significant moderate but positive correlation with the quality of life." can be revised to "Correlation analysis found moderate, but significant, association between academic performance and stress; better academic performance is associated with lower academic stress".

**Response:** The following amendment has been done and highlighted in the manuscript

“The Spearman correlation analysis found a moderate but significant, association between academic performance and stress; better academic performance is associated with lower academic stress, and a significant but moderate and positive association was found with a better quality of life”.

**Comment:** Table 2: Is the symbol "*" indicates significant differences between examined variables? If yes, shouldn't the p-value for DREEM inventory i.e., p =0.002 mark with "*"?

**Response:** The p-value for DREEM inventory i.e., p =0.002 is marked with "*" and highlighted in Table 2

**Comment:** Line 310-369 (Discussion): While the Discussion do address how the findings of current study compared to previous study, it definitely needs more work as it is lacking analytical insight from the authors. Also, the authors did not demonstrate how the findings in Phase 2 explains/relates to Phase 1's quantitative findings.

**Response:** The discussion section has been updated and changes have been highlighted in the manuscript.

**Comment:** Line 314 - 317: This information should be placed in the Results section.

**Response:** This has been addressed and highlighted in the manuscript

**Comment:** Line 394: In Line 177, the author mentioned that the study was conducted in a private institution in Peshawar, Pakistan. However, the author stated in Line 394 that the study involves two institutes. Please clarify which is the correct information.

**Response:** The study was conducted in two sister institutes. One is a medical college and the other one is a dental college. The sentences in the relevant sections have been rephrased to clarify. Relevant changes have been highlighted

**Comment:** Figure is not explained in the text.

**Response:** The figure has been referenced in the text and the added text has been highlighted

**Reviewer #3:**
**Comment:** The authors should follow the same sequence throughout the manuscript i.e. quantitative before qualitative in the abstract, results and discussion. The authors should be focused on their aims/objectives and let the results and discussion align with aims/objectives.

**Response:** The sequence has been updated carefully to align the results and the objectives of the study. The changes have been highlighted (L233-256 & 419-430)

**Abstract**
**Comment:** Let your results align with the aim of the study. Especially, the correlation aspect of the study. We are interested in the influence of stress and the educational environment on academic performance and QoL.

**Response:** The results have been aligned with the objectives of the study. The sentences have been rephrased for clarity and easy comprehension (L233-259)

**Comment:** Since your design is sequential-Exploratory Mixed-Methos as stated in your methods section, can you report your results in that sequence (Quantitative before qualitative).
**Response:** The sequence has been updated carefully to align the results and the objectives of the study. The changes have been highlighted

**Comment:** Can you make the abstracts' results a paragraph?

**Response:** The results are merged into one paragraph. The change has been highlighted in the manuscript.(L61-62).

**Introduction**
**Comment:** Can you add two or more statements on the association/relationship between the educational environment and quality of life?

The amendments have been done and changes are highlighted in the manuscript. (L130-134)

**Methodology**
**Comment:** Can you add a statement or more about the validity of the instruments? Let the readers know how you classified the parameters/variables of the instruments measured.
**Response:** The amendments have been done and changes are highlighted in the manuscript.

**Comment:** What informed the sample size of 10 for qualitative phase? Is it adequate?

**Response:** In a qualitative study, the primary focus is on obtaining in-depth and rich insights rather than generalizability to a larger population. The concept of data saturation is crucial in qualitative research, indicating the point at which new information or themes no longer emerge from additional data collection or analysis. By reaching data saturation, it implies that the collected information has reached a point of redundancy and that further interviews or data collection would not significantly contribute to the understanding of the research topic.

Hence, in our study, we deliberately chose to have a relatively small sample size of 10 participants. This decision was based on the established principle in qualitative research that a smaller sample can still provide valuable and comprehensive insights into the phenomenon under investigation. By conducting thorough and iterative interviews with these participants, we were able to delve deeply into their perspectives, experiences, and perceptions, allowing us to gain a thorough understanding of the research topic.

**Data analysis**
**Comment:** Three of the instruments used were on ordinal level of measurement, Spearman correlation will be appropriate instead of Pearson correlation.

**Response:** This issue has been addressed and the Spearman correlation has been incorporated. The changes are highlighted in the manuscript. (Table 3 L288-290)

**Comment:** The chi-square test measures association and not mean differences.

**Response:** The correction has been done and the change has been highlighted in the manuscript.

**Results**
**Comment:** Under the methodology above, you stated the age range was between 19 and 25 years and students older than 18 were included. Please reconcile why 18 to 25 years were included.
**Response:** This age range corresponds to the typical age range of students pursuing medical or dental education and ensures a homogeneous sample, facing challenges within this specific context. The age range of 18 to 25 years aligns with the nature and focus of the study, which specifically targets medical and dental students.

**Comment:** Table 2 showed results of association between the variables. Therefore, the two sentences cannot be said to be true based on table 2 (lines 200-202). If you are interested in mean differences, please run the appropriate test (ManWhiney U test for gender difference of stress, t-test or ANOVA for academic performance).

**Response:** This has been addressed and highlighted in the manuscript (L245-256).

**Comment:** Please reconstruct results of correlation for clarity. lines 203-209
**Response:** The amendments have been done and changes are highlighted in the manuscript. (L245-259).

**Discussion**
**Comment:** Focus on your aims and objectives in the discussion. Since your objective is to assess the effect of academic stress and educational environment on academic performance and QoL, let your discussion align with these. You can expand your discussion along this line especially, the correlation aspect of the study.
**Response:** The amendments have been done and changes are highlighted in the manuscript. (L419-423)

**Comment:** Your analysis does not support some of the results' discussion
**Response:** The amendments have been done and changes are highlighted in the manuscript.

(L243-256)

**Comment:** No mention of years of study influences academic stress in the results section. How come discussing it
**Response:** The statement has been added in the results section. (L243,244)
**Comment:** Let your conclusion be within the limit of your findings. Don't extrapolate nor draw inferences.
**Response:** The amendments have been done and changes are highlighted in the manuscript. (L461-470)

**Comment:** Figure
No reference to the figure in the manuscript.

**Response:** The reference to the figure with a brief explanation has been added and highlighted in the manuscript (L369-371)

**Reviewer # 4:** 
**Comment:** The title is too long and confusing, consider editing it

**Response:** Thank you for your kind suggestion. As it is a mixed method study and there are four variables covering major domains and the ethical approval letter also contains the same title changing the title will be a bit challenging at this point in time. However, considering your and other reviewers’ suggestions we have made amendments to the expression of results and discussion to make the text more comprehendible.

**Comment:** Ensure abbreviations are defined in their full meaning at their first use in the

abstract and the main article

**Response:** This issue has been addressed and changes have been highlighted in the manuscript.

L54,55 & L167,168

**Comment:** Line 144-147: Provide citation/reference for these statements

**Response:** Academic performance has varied definitions in the literature depending on the context in which research had been done. For our study, we have operationally taken annual professional exam results as a measure of academic performance. For clarity, the sentence has been rephrased and highlighted in the document. (L175, 176)

**Comment:** Line 172-173: …..“The interview guide was developed by following the protocol for
questionnaire development and was validated by the experts.”…..CITE THIS STATEMENT

**Response:** The statement has been cited and highlighted in the manuscript. (L209)

**Comment:** Line 175-182: Cite these statements

**Response:** The citations have been done and changes highlighted in the manuscript (L216-225)

**Comment:** Line 184-188: Cite these statements

**Response:** The citations have been done and changes highlighted in the manuscript (L227-231)
