# Supplementary material for: Effect of academic stress, educational environment on academic performance & quality of life of medical & dental students; gauging the understanding of health care professionals on factors affecting stress: A mixed method study
Source: PLoS One. 2023 Nov 28;18(11):e0290839. doi: 10.1371/journal.pone.0290839 (PMC10683982; doi:10.1371/journal.pone.0290839)
Supplement: Attachment — Submitted filename: Rebuttal letter Response to Reviewers (140823).docx [file pone.0290839.s005.docx]

**Rebuttal Letter:**

**Article No: [PONE-D-23-12972] “Effect of academic stress, educational environment on academic performance & quality of life of medical & dental students; Gauging the understanding of health care professionals on factors affecting stress: A mixed method study”**

Dear Editor,

Gratitude again to the esteemed reviewers for their reviews and generous consideration in advancing the study further. We have diligently endeavoured to incorporate all the recommendations put forth by the reviewers in our revised version of the manuscript. Below, you will find a comprehensive and systematic response to each of the comments of the reviewers.

**Please note that the line numbers indicated in the responses correspond to the submitted document labelled “Revised manuscript with track changes”.

**Reviewer #2:**

**Comment:1**

The authors have addressed the previous comments except for the following:

1. There are still no information provided on the scoring method to categorise the following:

a) WHOQOL (Better vs. Poor)
b) DREEM Inventory (Excellent vs. Positive vs. Plenty of problems)

c) Academic stress (High vs. No)

**Response:** The amendments have been done and changes are highlighted in the manuscript.

The following lines have been revised in the manuscript along with improvement in language.

**WHOQOL**

**LINE** **172-178:** Cronbach`s alpha was 0.85, 0.83, 0.62, and 0.81, respectively, for the physical, psychological, social, and environmental domains, and 0.92 for the total scale. The level of internal consistency was acceptable to good [15]. Those who scored more than the mean score of 61.97 were considered to have better QoL and those less than 61 had Poor QoL.

**DREEM**

**LINE 184-193:**  The 50-item Dundee Ready Education Environment Measure (DREEM) questionnaire assesses the quality of the educational environment, and responses are rated on a five-point Likert scale, spanning from 0, representing strong disagreement, to 4, signifying strong agreement. The comprehensive cumulative score encompasses values from 0 to 200. Within the 50-item scale, 9 items are negatively stated and necessitate subsequent reverse scoring. The evaluation of students' environmental perception was stratified into the following categories: an aggregate score between 0 and 50 indicated a very poor perception of the environment, a range of 51 to 100 indicated plenty of problems, the interval of 101 to 150 signifies a preponderance of positive aspects relative to negative facets, and a score falling within 151 to 200 was characterised as indicative of an excellent environment.

**Academic Stress Scale:**

**LINE 212-215:**  Those who scored more than the mean score of 67.13 were considered to have high academic stress and those less than the mean score of 67 had no academic stress. Overall, the higher scores on the mean average indicated more academic stress.

**Comment:2**

Please include a justification for sample size estimation of qualitative study in the text.

**Response:** Thank you for your comment. Actually, the researchers identified the faculty members who fulfilled the eligibility criteria and communicated with the faculty member before conducting the interviews. After each interview, the transcripts were analysed. Once the researchers identified that no new insights are coming up and the same information is being generated (i.e. data saturation is reached), they stopped further interviews. Researchers reached the **data saturation point after 10 interviews,** and it was taken as sample size. This technique of determining the sample size is well established in the literature [(Hennink M, Kaiser BN. Sample sizes for saturation in qualitative research: A systematic review of empirical tests. Social science & medicine. 2022 Jan 1;292:114523.)](https://scholar.google.com/scholar?hl=en&as_sdt=0%2C5&q=Sample+sizes+for+saturation+in+qualitative+research%3A+A+systematic+review+of+empirical+tests&btnG=)

To clarify this point, the following amendments have been done and changes are highlighted in the manuscript.

**LINE 221-229:**  Faculty members who fulfilled the eligibility criteria were approached to consent to one-to-one interviews. The principal investigator conducted in-depth interviews with healthcare professionals in order to comprehensively investigate their viewpoints concerning the determinants contributing to academic stress among students. Each interview session lasted between 40 to 60 minutes, with the interviews continuing until the point of data saturation, whereby no novel insights were forthcoming. The saturation point was attained upon the completion of 10 interviews. The verbatim content of the audio-recorded interviews was transcribed and subsequently subjected to validation from the interviewees.

**Comment:3**

The content of Discussion is limited to how findings of current study compared to literature. Thus, it definitely needs more work as it is lacking analytical insight from the authors. Please demonstrate how the findings in Phase 2 explains/relates to Phase 1's quantitative findings.

**Response:** The amendments have been done and changes are highlighted in the manuscript.

**LINE 456-458:**  The qualitative phase supplemented the quantitative results by offering deeper insights into the factors influencing academic stress, educational environment, and their impact on academic performance and quality of life among medical and dental students.

**LINE 478-483:** The qualitative findings in our study endorse the challenging educational environment experienced by medical and dental students, impacting academic stress and performance, and are supported by the literature [8, 12, 42]. Psychological support, individual differences, social and family relationships, and adjustment issues in shaping students' experiences and outcomes are the influencing factors well comprehended in the literature [4,10,12].

**Comment:4**

Language revision is highly recommended.

**Response:** The amendments have been done and changes are highlighted in the manuscript

Language revisions have been done at various places in the document while addressing the comments of worthy reviewers and also while proofreading the document for clarity. The changes have been highlighted in the following lines in the manuscript:

**LINE 45-47:**  Our study aims to ascertain the effect of academic stress and the educational environment on the QoL and academic performance of medical and dental students, encompassing the perspectives of both students and healthcare professionals.

**LINE 184-193:**  The 50-item Dundee Ready Education Environment Measure (DREEM) questionnaire assesses the quality of the educational environment, and responses are rated on a five-point Likert scale, spanning from 0, representing strong disagreement, to 4, signifying strong agreement. The comprehensive cumulative score encompasses values from 0 to 200. Within the 50-item scale, 9 items are negatively stated and necessitate subsequent reverse scoring. The evaluation of students' environmental perception is stratified into the following categories: an aggregate score between 0 and 50 indicates a very poor perception of the environment, a range of 51 to 100 indicates plenty of problems, the interval of 101 to 150 signifies a preponderance of positive aspects relative to negative facets, and a score falling within 151 to 200 is characterised as indicative of an excellent environment.

**LINE 200-203:**  DREEM is a widely accepted and universally validated tool, demonstrating an internal consistency of 0.86 and a test-retest reliability of 0.595 (p < 0.001). Previous studies utilizing Confirmatory Factor Analysis have affirmed the good model fit for the five-factor structure of DREEM-50 [16,17].

**LINE 221-229:**  Faculty members who fulfilled the eligibility criteria were approached to consent to one-to-one interviews. The principal investigator conducted in-depth interviews with healthcare professionals in order to comprehensively investigate their viewpoints concerning the determinants contributing to academic stress among students. Each interview session lasted between 40 to 60 minutes, with the interviews continuing until the point of data saturation, whereby no novel insights were forthcoming. The saturation point was attained upon the completion of 10 interviews. The verbatim content of the audio-recorded interviews was transcribed and subsequently subjected to validation from the interviewees.

**LINE 171-178:**  Cronbach`s alpha was 0.85, 0.83, 0.62, and 0.81, respectively, for the physical, psychological, social, and environmental domains, and 0.92 for the total scale. Previous studies employing Confirmatory Factor Analysis on the WHOQOL-BREF have documented good to excellent reliability properties and better performance on initial validity tests [15].

**LINE 256-261:**  Our study involved 500 students from a medical and a dental college with a participation rate of 80% (n=440). The average age of the participants was 22.24±1.5 years, with an age range of 18 to 25 years. Of the participants, 51% were male (n=224), and 62.3% (n=274) indicated they lived in a nuclear family setup. The internal consistency of the instruments was strong, with a Cronbach's alpha reliability coefficient of 0.877 for the DREEM inventory, 0.939 for the Academic Stress Scale, and 0.895 for the WHOQOL-BREF.

**LINE 398-400:** Fig 1, illustrates the sequential explanatory design employed in the study. The qualitative input from healthcare professionals who were the counsellors of the students enriched the interpretation of quantitative findings.

**LINE 456-458:** The qualitative phase supplemented the quantitative results by offering deeper insights into the factors influencing academic stress, educational environment, and their impact on academic performance and quality of life among medical and dental students.

**LINE 476-483:** Nursing students are integral members of the healthcare sphere and share the same stressful environment as medical institutions and this implies a plausible parallelism in the outcomes. The qualitative findings in our study endorse the challenging educational environment experienced by medical and dental students, impacting academic stress and performance, and are supported by the literature [8, 12, 42]. Psychological support, individual differences, social and family relationships, and adjustment issues in shaping students' experiences and outcomes are the influencing factors well comprehended in the literature [4,10,12].

**Reviewer #3:**

The authors have addressed most of the comments previously highlighted. The manuscript has improved substantially. A few comments are yet to be addressed.
**Comment:1**

Abstract: You have omitted the clear aim(s) in the abstract. You only provided background and the need.

**Response:** The amendments have been done and changes are highlighted in the manuscript. Due to the work count limit of the abstract, the need and aim of the study have been summarized in one sentence as follows:

**LINE 45-47:** Our study aims to ascertain the effect of academic stress and the educational environment on the QoL and academic performance of medical and dental students, encompassing the perspectives of both students and healthcare professionals.

**Comment:2**

Methods "Can you add a statement or more about the validity of the instruments? Let the readers know how you classified the parameters/variables of the instruments measured." You have not addressed these comments.
**i. Please provide statements about the validity of each instrument you described.**

**Response:** Following amendments have been made in the manuscript and highlighted

**World Health Organization, Quality of Life Scale: (WHOQOL-BREF):**

**LINE 172-178:** Cronbach`s alpha was 0.85, 0.83, 0.62, and 0.81, respectively, for the physical, psychological, social, and environmental domains, and 0.92 for the total scale. Previous studies employing Confirmatory Factor Analysis on the WHOQOL-BREF have documented good to excellent reliability properties and better performance on initial validity tests [15].

**DREEM**

**LINE 200-203:**  DREEM is a widely accepted and universally validated tool, demonstrating an internal consistency of 0.86 and a test-retest reliability of 0.595 (p < 0.001). Previous studies utilizing Confirmatory Factor Analysis have affirmed the good model fit for the five-factor structure of DREEM-50 [16,17].

**Academic Stress Scale:**

The 40-item academic stress scale validity studies have not been found in the literature; however, internal consistency of the scale has been demonstrated in various studies and our study also.

The following description has been added and highlighted in the literature:

**LINE 210-211:** The Academic Stress Scale has a good internal consistency score with a Cronbach alpha of 0.70 [18].
**ii. It is important to indicate mean cutoff mark for better QoL in the description of instrument under methods for readers understanding. In your results, you mentioned the percentage of participants who had better QoL**.

**Response:** Following amendments have been made in the manuscript and highlighted

**LINE 176-178:**  Those who scored more than the mean score of 61.97 were considered to have better QoL and those less than 61 were categorized as having poor QoL.

**iii. It important to indicate mean cutoff mark for having academic stress in the description of instrument under methods for readers understanding. In your results, you mentioned the percentage of participants who had academic stress.**

**Response:** Following amendments have been made in the manuscript and highlighted

**LINE 212-215:**  Those who scored more than the mean score of 67.13 were considered to have high academic stress and those less than the mean score of 67 had no academic stress. Overall, the higher scores on the mean average indicated more academic stress.
